# Value of Scarlet Red Ointment in Granulating Wounds

**Published:** 1913-12

**Authors:** T. C. Davison

**Affiliations:** Atlanta, Ga.; Associate Professor of Surgery Atlanta College of Physicians and Surgeons; Visiting Surgeon to the Georgia Baptist Hospital, and Associate Surgeon to Grady Hospital


### VALUE OF SCARLET RED OINTMENT IN GRANULATING WOUNDS.*

By T. C. Davison, M. D., Atlanta, Ga.

Associate Professor of Surgery Atlanta College of Physicians and Surgeons; Visiting Surgeon to the Georgia Baptist Hospital, and Associate Surgeon to Grady Hospital.

Scarlet Red is an anilin dye, a dark red powder, and was formerly used exclusively for technical purposes. Chemically it is a sodium salt of amido-azo-benzene disulphonic acid azo-betanapthol.

Scarlet Red medicinal (Biebrich) is used in making Scarlet Red Ointment (Parke, Davis & Company). It is used in the form of an ointment of various strengths from 4% to 10% as the powder itself is more or less irritating. My experience has been that the stronger preparations are irritating to the skin surrounding the wound. I therefore use 4% or 8%, which, when applied to granulating wounds, stimulates the formation of epithelium, thereby proving very valuable in the treatment of indolent ulcers.

It closes breaks in the continuity of the skin, not by hastening the formation of scar tissue, but by actual cell proliferation from the epithelial edges of the wound, which thus results in the development of normal skin.

This ointment has been found useful in treating ulcers following burns, varicose ulcers, syphilitic ulcers, bedsores, tubercular ulcers, x-ray burns, and ulcers resulting from infection and trauma. I have also used it beneficially in cases of skin grafts forty-eight hours old.

MODE OF APPLICATION.—The most successful method of application I find is first to cleanse the wound thoroughly with a solution of boric acid or other mild antiseptic. It isn't best to use irritating applications unless especially indicated. One may, however, apply hydrogen peroxide if pus is present, or silver nitrate to control exuberant granulations. Tincture of iodine, 5 to 10% in alcohol, and also Turpedine are excellent applications to infected wounds to kill the infection. After the foregoing preparation, a thin layer of ointment is spread on gauze large eniugh to cover the wound. It is best to avoid the surrounding skin owing to possible irritation, especially when using the 8 to 10% ointment, or if the application is left on for several days. After applying the ointment cover with sterile gauze and bandage according to indications.

STRENGTH.—This preparation, as previously mentioned, is marketed in various strengths, some physicians recommending the use of an 8 or 10% ointment, alternating daily with some bland application, as boric acid ointment, to prevent undue irritation, and the breaking down of granulations already formed. I have found it better, however, to use the weaker 4 or 5% ointment as one does not have to be so careful about alternating with other aplpications. The ulcer should be dressed every forty-eight hours or oftener if especially indicated. It is not advisable to put on the ointment too thick, as it is then more likely to irritate.

Scarlet Red salve is not antiseptic, therefore must not be depended on to kill out the infection. I first try to get rid of the specific microorganisms; then employ the scarlet salve to hasten granulation. Or it may be combined with antiseptics such as boric acid, iodoform, balsam of Peru, blue ointment, etc. The Scarlet Red ointment has a drying effect and may produce a scaly condition of the skin after healing, but this is easily overcome by oily applications.

I have had excellent opportunity to experiment clinically with this remedy on all kinds of ulcers in the out-door service at the Atlanta College of Physicians and Surgeons, where I have been using it for the past two years. This class of patients, as a rule, is very unsatisfactory, as they have to work, and cannot give the parts the rest they should. Neither can they be seen regularly, usually twice a week, often but once a week and at tims less often; but even under these great disadvantages I can report wonderful success in many cases, having relieved varicose ulcers of several years standing in a few weeks or a few months. They have since been operated upon which means a permanent cure for them.

Syphilitic ulcers heal much more promptly under Scarlet Red treatment. I usually alternate with blue ointment in these cases. Venereal ulcers and incised buboes heal promptly after applying the proper antiseptic for the specific infection and then the scarlet salve. Ulcers whose surface is covered with necrotic tissue or abundant purulent secretion are unfavorable for the ointment and in these cases it is necessary first to get the ulcer clean if possible, and then to apply the salve to stimulate the rapid formation of epithelium. I do not claim that it is a "cure-all," but do say that healing is essentially hastened, and that many indolent ulcers that have been haunting your office will be healed up in a surprisingly short time.

906 Empire-Life Bldg.

